# Investigating Emotional Intelligence: A Cross-Sectional Study of Pune’s Postgraduate Medical Scholars

**DOI:** 10.7759/cureus.74424

**Published:** 2024-11-25

**Authors:** Gayatri R Nair, Sai Mahesh Vajjala, Akash Nagar, Akhil R, Samyukta Nair, Diksha Aghi, Shubhangini Mishra, Jyotsna Ahuja

**Affiliations:** 1 Community Medicine, Dr. D. Y. Patil Medical College, Hospital and Research Centre, Dr. D. Y. Patil Vidyapeeth (Deemed to Be University), Pune, IND; 2 Medicine, Dr. D. Y. Patil Medical College, Hospital and Research Centre, Dr. D. Y. Patil Vidyapeeth (Deemed to Be University), Pune, IND

**Keywords:** college, doctors, emotional intelligence, medical education, postgraduates

## Abstract

Introduction

Emotional intelligence (EI), which encompasses the ability to perceive, understand, and manage emotions, is crucial for effective human interaction. In healthcare, especially in medicine, compassion and empathy are prioritized qualities associated with enhanced patient outcomes, increased patient compliance, and overall improved healthcare experiences. This study focused on postgraduate medical students to assess their EI levels and identify influencing factors.

Methods

This cross-sectional study aimed to evaluate the EI of postgraduate students at a medical college in Pune. Participants, who willingly participated, engaged in face-to-face interviews where they completed a questionnaire addressing sociodemographic information, workload, stress, and Schutte's Emotional Intelligence Test. Data analysis, conducted using MedCalc and Epi Info, presented qualitative variables as frequencies and quantitative data as mean (SD) or median (IQR). Statistical tests were used to identify the associations.

Results

Involving 139 postgraduates, our study found a mean EI score of 123.48 (12.30). The majority, 97(69.78%), displayed normal scores, 23 (16.55%) scored low, and 19 (13.67%) scored high. Males had higher EI scores. EI increased slightly from the first year to the final year. No significant associations were found with marital status or medical specialty; however, those facing recent emotional trauma exhibited higher levels.

Conclusion

EI exhibited no significant associations with age, marital status, residential status, year of study, duty hours, stress, or specialty. However, EI demonstrated significant association with male gender and those who faced emotional trauma.

## Introduction

Emotional intelligence (EI), as defined by Salovey and Mayer (1990), is a form of social intelligence that encompasses the capacity to perceive and comprehend one's own and others' emotions, distinguish between them, and apply this understanding to manage their thoughts and behaviors effectively. In their Four Branch Model, Salovey and Mayer outlined EI as proficiency in (a) recognizing emotions, (b) leveraging emotions to enhance cognitive processes, (c) comprehending emotions, and (d) effectively managing emotions [1]. Later Goleman (1995) suggested that EI consists of five components: understanding one’s own emotions (self-awareness), effectively managing them, motivating ourselves, recognizing emotions in others (empathy), and handling relationships [2]. According to Goleman, EI is more important than cognitive intelligence for success in life [3].

EI enables individuals to understand and regulate their emotions, facilitating effective human interaction. It also helps them empathize and respond to the emotions of others, contributing to successful interpersonal relationships [4]. EI is not just essential for overall well-being and life satisfaction but also contributes to about 80% of intelligence quotient and academic achievements, and reduces violent behavior, negative emotions, and substance abuse [5,6].

A growing body of research suggests that EI may be important for both professional practice and mental health in medicine, nursing, and other healthcare disciplines [3]. Compassion and empathy have long been coveted qualities in a physician. Empathic doctors are better at obtaining a thorough history, giving emotional support, making a correct diagnosis, and getting patients to comply with their recommended treatments. Patients experience a sense of well-being when their doctor exhibits empathy and compassion, which accelerates their recovery [4]. It is assumed that those with high EI levels have stronger interpersonal and communication abilities, and thus, have a favorable effect on work satisfaction, reducing occupational stress, burnout, and providing quality care [7]. Violence against physicians and other healthcare workers has recently increased, particularly in India and other low- and middle-income nations. Most of these incidents were caused by the stress of the overworked medical staff. They can also be linked to the medical community's general lack of EI, which aggravates this problem [4].

In an effort to consider an applicant's interpersonal abilities, several medical schools are now using EI evaluation as part of the selection process [8]. In order to produce sensitive and compassionate doctors for the future, it is important to teach students EI abilities as part of their medical curriculum. The substance of the medical curriculum is so dense that there is frequently little time left for skill development. The majority of these soft skills, including effective communication, EI, and empathy, are learned subconsciously through the observation of seniors at the bedside and in outpatient clinics. The National Medical Commission is focusing on the AETCOM module (attitude, ethics, and communication module) in order to impart education on communication skills, empathy, EI, and ethics. Postgraduate students are future specialists who are more involved in patient care. This study aimed to evaluate EI levels among postgraduate medical students and identify the factors that contribute to it.

## Materials and methods

This cross-sectional study was conducted among postgraduate students in various departments of a medical college in Pune from September to October 2022, following approval from the Institutional Ethics Committee. All postgraduate students who willingly consented to participate were included, and participant selection was facilitated through convenience sampling. Informed consent was obtained from all the study participants at the outset. A questionnaire administered via face-to-face interviews encompassed inquiries about sociodemographic details, workload, stress levels, and the Schutte Self-Report Emotional Intelligence Test questionnaire.

EI was assessed using Schutte's Self-Report Emotional Intelligence Test questionnaire, a validated 33-item self-report tool based on the Salovey and Mayer Emotional Intelligence model developed by Schutte et al. [9]. Each question utilized on a 5-point Likert scale, ranging from "strongly disagree" to "strongly agree," with scores ranging from 33 to 165.

A study conducted in Delhi by Ravikumar et al. evaluated EI among postgraduate medical students. The mean EI score was reported as 124.4 ± 12.8 [10]. To estimate the mean EI, considering 12.8 standard deviation and an absolute precision of 2.5, at 95% confidence, the minimum sample size was calculated to be 105. Notably, the study included 139 participants. The software used for calculation was WinPepi version 11.38 (J.H. Abramson, Brixton Health, London, UK), alternatively formula used for calculation was (n=(Z\alpha)^2 * (SD)^2/ (d)^2\). 

Data entered into Excel sheet was analyzed using software like MedCalc 18.2.1 and Epi Info 7.2.5.0.

Categorical variables were presented as frequencies and percentages. Continuous data following a normal distribution were summarized using mean and SD, whereas non-normally distributed data were represented by median and IQR. Shapiro-Wilk test was used to assess normal distribution. The association between various factors affecting EI scores was analyzed using statistical tests such as the Independent T-test for normally distributed variables and the Mann-Whitney U Test for non-normally distributed variables. In instances where variables consisted of more than two groups and displayed a normal distribution, analysis of variance (ANOVA) was employed; alternatively, for non-normally distributed variables, the Kruskal-Wallis test was applied. In all tests, statistical significance was set at P < 0.05. Bonferroni correction was not applied. 

## Results

A total of 139 postgraduates from various departments of a medical college in India participated in the study. The age of the participants ranged from 23 to 39 years, with a mean age of 27.22 (2.76) years. The majority of the participants, 88 (63.31%), belonged to the age group of 26-30 years. Among the participants, 80 (57.55%) were females, while 59 (42.45%) were males.

Out of 139 total study participants, 111 (79.86%) participants were residing away from their families, with 97 (69.78%) residing in hostels and 14 (10.07%) staying as paying guests. A total of 111 (79.86%) participants were single, whereas 28 (20.14%) were married. Of the respondents, 90 (64.75%) were from clinical departments and 49 (35.25%) were from paraclinical/pre-clinical departments. 103 (74.10%) participants were in their first year of postgraduate study and 36 (25.90%) were in their final year.

103 (74.10 %) participants reported working overtime. Among the study participants, 64 (46.04%) had a weekly workload exceeding 96 hours, while 57 (41.01%) had duty hours between 48 and 96 hours per week. However, a smaller subgroup of 18 participants (12.95%) reported duty hours of less than 48 hours per week. Additionally, 74 (53.24%) participants did not have any off-days in the week, and 76 students (54.68%) reported experiencing constant stress.

The mean score of EI among participants was 123.48 (12.30), with a minimum score of 91 and a maximum score of 151. Scores below 111 or above 137 are considered outside the typical range and unusually low or high, respectively. Among the study participants, 97 (69.78%) had normal EI scores, 23 (16.55%) had low EI scores, and 19 (13.67%) had high EI scores. Description of other demographic and work related variables of the study population is mentioned in Table 1.

**Table 1:** Descriptives of demographic details and work-related characteristics of the study population Total: 139 participants

Factor	N (%)
1.Age	
21-25	38 (27.34%)
26-30	88 (63.31%)
31-35	9 (6.47%)
36-40	4 (6.47%)
2.Residence	
Hostel	97 (69.78%)
Staying at a relatives place or with local guardian	2 (1.44%)
Paying guest	14 (10.07%)
Staying with family	26 (18.71%)
3.Speciality	
Medical	110 (79.14%)
Surgical	29 (20.86%)
4.Number of night duty per week	
More than 3 night duties	29 (20.86%)
Less than or equal to 3 night duties	110 (79.14%)
5.Number of emergency duty per week	
Less than or equal to 2	107 (76.98%)
More than 2	32 (23.02%)
6.Number of duty off days per week	
0	74 (53.24%)
1	62 (44.60%)
2	3 (2.16%)
7.Do you feel that you are always in stress?	
Yes	76 (54.68%)
No	63 (45.32%)
8.Are you taking any psychiatric medications?	
yes	2 (1.44%)
no	137 (98.56%)
9.Have you experienced death of a close family member in the recent past?	
Yes	52 (37.41%)
No	87 (62.59%)
10.Have you experienced any relationship breakup in the recent past?	
Yes	41 (29.50%)
No	98 (70.50%)

EI showed significant associations with gender (p=0.0305) and individuals who experienced the death of a close family member (p≤0.0001). However, factors such as age, marital status, residential status, specialty, year of study, workload, and stress did not exhibit notable associations. Table 2 states the association of various factors with EI.

**Table 2:** Association of various factors with EI *Association between three factors tested by one-way analysis of variance (ANOVA) ** Association between two factors tested by t-test (Independent t-test) EI, emotional intelligence

Variable		N	Mean of EI score (SD)	Min - max value of EI score	Significance
1.Age (in years)	21-25	38	122.53 (12.16)	91.00-147.00	F (3.135)=0.460, *p=0.711
	26-30	88	123.96 (12.42)	97.00-150.00	
	31-35	9	120.78 (11.50)	101.00-133.00	
	36-40	4	128.25 (15.52)	117.00-151.00	
2.Gender	Female	80	121.55 (12.58)	91.00-150.00	t=2.186, df=137, **p=0.0305
	Male	59	126.10 (11.50)	102.00-151.00	
3.Marital status	Married	28	121.89 (13.13)	91.00-151.00	t=0.764, df=137, **p=0.4462
	Single	111	123.88 (12.11)	97.00-150.00	
4.Residence	Hostel	97	122.70 (11.78)	91.00-150.00	F (3.135)=1.626, *p=0.186
	Staying at a relatives place or with local guardian	14	129.36 (15.93)	98.00-151.00	
	Paying guest	2	114.00 (8.49)	108.00-120.00	
	Staying with family	26	123.96 (11.75)	101.00-148.00	
5.Speciality	Medical	110	122.94 (12.04)	97.00-151.00	t=1.019, df=137, **p=0.3101
	Surgical	29	125.55 (13.26)	91.00-150.00	
6.Year of study	First Year	103	122.99 (12.80)	91.00-151.00	t=0.796, df=137, **p=0.4273
	Third Year	36	124.89 (10.77)	101.00-148.00	
7.Total hours of duty per week?	<48 hours	18	124.33 (9.18)	105.00-143.00	F (2.136)=0.199, *p=0.819
	48-96 hours	57	122.70 (12.94)	97.00-151.00	
	>96 hours	64	123.93 (12.60)	91.00-150.00	
8.Number of duty off days per week?	0	74	123.16 (12.29)	91.00-150.00	F (2.136)=0.060, *p=0.942
	1	62	123.89 (12.54)	98.00-151.00	
	2	3	123.00 (10.82)	111.00-132.00	
9.Number of night duty per week?	>3	29	125.31 (12.76)	105.00-151.00	t=0.899, df=137, **p=0.3701
	≤3	110	123.00 (12.19)	91.00-150.00	
10.Number of emergency duty per week?	Less than or equal to 2	107	122.57 (12.24)	91.00-150.00	t=1.608, df=137, **p=0.1102
	More than 2	32	126.53 (12.18)	105.00-151.00	
11.Do you feel that you are always in stress?	Yes	76	123.32 (12.74)	91.00-150.00	t=0.174, df=137, **p=0. 8618
	No	63	123.68 (11.85)	98.00-151.00	
12.Are you taking any psychiatric medications?	Yes	2	111.00 (15.56)	100.00-122.00	t=1.451, df=137, **p=0.1490
	No	137	123.66 (12.22)	91.00-151.00	
13.Have you experienced any relationship breakup in the recent past?	Yes	41	123.39 (14.08)	91.00-151.00	t=0.0567, df=137, **p=0.9549
	No	98	123.52 (11.55)	97.00-150.00	
			Median (IQR) of EI score		
14.Have you experienced death of a close family member in the recent past?	Yes	52	133.00 (130.00 to 140.00)	108.00-151.00	U=245.00, p≤0.0001; Mann-Whitney U test done for association

## Discussion

EI is a crucial human quality involved in fostering the patient-physician relationship. During a period of increased competition for patient adherence, clinicians who are more cognizant of their patients' feelings are more effective in treating them than their less observant co-workers.

The mean EI score of 123.48 (12.30) aligns with comparable studies done using the same questionnaire, suggesting a consistent level of gross emotional functioning among medical students [10-12]. It can possibly be attributed to shared educational backgrounds, uniform curriculum, shared experiences, extensive clinical exposure, and stringent admission procedures in medical schools, which place a strong emphasis on both interpersonal and intellectual competence. This collectively contributes to the cohesive evolution of students’ EI.

The highest mean EI score, recorded at 128.25 (15.52) within the 36-40 age range, suggests a potential connection between enhanced maturity and heightened empathy. This underscores the idea that age and life experiences may collectively contribute to an individual's increased sensitivity to both personal and other sentiments. However, the aggregate EI scores across age groups showed no significant differences, aligning with a study done by Harrod et al. but contradicting findings from studies conducted in Delhi, Kerala, and Central India [10,13-15].

The gender-based comparison of EI revealed a statistically significant association, with male postgraduate students exhibiting higher mean scores, consistent with findings in various other studies [15-17]. However, in contrast to this pattern, a study in Pakistan and Pondicherry reported higher EI scores among females [18,11]. One possible explanation for these variations is that men may demonstrate elevated levels of self-awareness, assertiveness, independence, and situational management [17]. On the other hand, studies conducted by Sundararajan et al. among Chennai-based medical students found that gender had no discernible impact on EI scores [4,10].

The EI scores exhibited minimal variation based on marital status, mirroring findings from other studies [10,18]. This consistency may be attributed to the understanding that EI is primarily shaped by multifaceted factors, such as personality, life experiences, and individual development, rather than being significantly linked to one's marital status.

The lowest mean EI score of 114.00 (8.49) in individuals staying as paying guests may be attributed to their solitary living arrangements, causing a potential negative impact on EI due to reduced social interactions and limited support systems.

Our study reveals a slight upward trend in EI scores between first- and third-year postgraduate students, although not statistically significant, aligning with similar observations in the study by Ravikumar et al. [10]. The progression of EI during academic advancement is attributed to active engagement in intricate real-world situations, including clinical rotations, internships, and hands-on experience. This contrasts with results from Nitin Joseph's study, highlighting divergent findings [19].

No significant association emerged between EI scores and medical specialty, which is consistent with the findings of a study conducted in Delhi [10]. This alignment highlights the shared nature of medical education, where uniform training and similar challenges emphasize the critical role of effective communication and empathy across various specialties [20].

Contrary to some studies, our findings revealed no impact of heavy workload on EI [15,10]. This divergence may be attributed to effective stress management, strong interpersonal connections, and comprehensive training among our participants.

In contrast to the study done by Ravikumar et al. proposing a negative association between EI and stress, our study revealed no significant difference in EI scores between stressed and non-stressed individuals, echoing findings from a Pondicherry study [10,11]. This discrepancy may be indicative of individual variations or the more effective stress management strategies employed by our study participants.

College students frequently face mental health challenges, with some relying on psychiatric medication. Research underscores the protective role of EI (EQ) against issues like stress, worry, and depression [21]. Our study corroborates these findings, indicating lower EI levels among individuals using psychiatric medication.

Individuals who experienced emotional trauma, such as recent family loss, exhibited a higher level of EI, paralleling the findings of David Tuck et al. [22]. Those with mental or physical trauma often excel in handling negative emotions, which is reflected in their elevated EI. Citing Faye et al.'s research, individuals facing home-related challenges displayed increased self-awareness and sensitivity, potentially impacting patient management and bedside behavior [23].

The use of a self-reported questionnaire may introduce reporting and recall bias, as participants might provide socially desirable responses. Additionally, the absence of a qualitative component limits a more comprehensive understanding of EI, which is dynamic and influenced by time and context. The sample was restricted to first- and third-year postgraduate medical students due to a NEET exam delay, and the use of convenience sampling may limit the generalizability of the findings to a broader population.

## Conclusions

In our study, most participants demonstrated normal EI scores. Age, marital status, residential status, year of study, duty hours, stress, and specialty showed no significant associations with EI. Despite this, individual variations persist and may evolve over time. Notably, EI exhibited significant associations with gender and experiences of a close family member's death. The enhancement of EI stands as a potential avenue for improving patient care and addressing the communication gap between doctors and patients.

Recognizing the pivotal link between EI and overall well-being, educational institutions and healthcare systems should prioritize stress management and EI development. Integrating Competency-Based Medical Education (CBME), Attitude, Ethics, and Communication (AETCOM), and Community-Oriented Medical Education (COME) will contribute to nurturing emotionally intelligent and resilient postgraduate medical professionals in India. This holistic approach is essential for elevating patient care, fostering professional satisfaction, and ensuring the well-being of medical residents.
